# Modulation effect of sulfated polysaccharide from *Sargassum fusiforme* on gut microbiota and their metabolites *in vitro* fermentation

**DOI:** 10.3389/fnut.2024.1400063

**Published:** 2024-05-01

**Authors:** Long Jiang, Chen Song, Chunqing Ai, Chengrong Wen, Shuang Song

**Affiliations:** SKL of Marine Food Processing and Safety Control, National Engineering Research Center of Seafood, Collaborative Innovation Center of Seafood Deep Processing, National and Local Joint Engineering Laboratory for Marine Bioactive Polysaccharide Development and Application, Liaoning Key Laboratory of Food Nutrition and Health, School of Food Science and Technology, Dalian Polytechnic University, Dalian, China

**Keywords:** *Sargassum fusiforme*, sulfated polysaccharide, fermentation, Bacteroides, gut microbiota

## Abstract

The present study demonstrated the digestion behavior and fermentation characteristics of a sulfated polysaccharide from *Sargassum fusiforme* (SFSP) in the simulated digestion tract environment. The results showed that the molecular weight of two components in SFSP could not be changed by simulated digestion, and no free monosaccharide was produced. This indicates that most of SFSP can reach the colon as prototypes. During the fermentation with human intestinal flora *in vitro*, the higher-molecular-weight component of SFSP was utilized, the total sugar content decreased by 16%, the reducing sugar content increased, and the galactose content in monosaccharide composition decreased relatively. This indicates that SFSP can be selectively utilized by human intestinal flora. At the same time, SFSP also changed the structure of intestinal flora. Compared with the blank group, SFSP significantly increased the abundance of Bacteroidetes and decreased the abundance of Firmicutes. At the genus level, the abundances of *Bacteroides* and *Megamonas* increased, while the abundances of *Shigella*, *Klebsiella*, and *Collinsella* decreased. Moreover, the concentrations of total short-chain fatty acids (SCFAs), acetic, propionic and n-butyric acids significantly increased compared to the blank group. SFSP could down-regulate the contents of trimethylamine, piperidone and secondary bile acid in fermentation broth. The contents of nicotinic acid, pantothenic acid and other organic acids were increased. Therefore, SFSP shows significant potential to regulate gut microbiota and promote human health.

## 1 Introduction

Bioactive polysaccharides derived from various natural resources have been proven to have prebiotic properties (1–3). In recent years, the fermentation of polysaccharides by gut microbiota has received increasing attention due to its benefits on host health, such as preventing cancer (4), improving lipid metabolism (5), and affecting gut microbiota (6). As is well known, the biological activity of bioactive components is closely related to the degradation and absorption in the digestive system. Some reports have shown that bioactive polysaccharides can go through the gastrointestinal tract and reach the distal end of the gastrointestinal tract. It has been well documented that various of indigestible polysaccharides could be degraded by gut microbes, such as species from *Bacteroides* (7). Moreover, sugars released from indigestible polysaccharides in the fermentation by some intestinal bacteria often support microbial growth and survival in the gut, and further regulate the gut microbial metabolism (8). Notably, the metabolites of gut microbiota play an important role in improving colon health, and further benefit the host health. In addition to short chain fatty acids (1, 5, 9), other microbial metabolites in gut could also regulate the host physiological state, such as trimethylamine (10) and secondary bile acids (11). Thus, these indigestible polysaccharides can alter the structure and metabolism of gut microbiota to promote the health of host through gut-brain axis, gut-liver axis, gut-lung axis, etc.

*Sargassum fusiforme* is a brown alga belonging to the Sargasaceae family and it is used as a drug in traditional Chinese medicine to treat diseases, such as tumor, scrofula, edema, beriberi, and chronic bronchitis (12, 13). Nowadays, large-scale aquaculture of *S. fusiforme* has been carried out in the coastal areas of Zhejiang and Fujian in China, with an annual output of nearly 30 thousand tons. The chemical composition of sulfated polysaccharides from *S. fusiforme* (SFSP) extracted by different methods varies slightly, but they mainly contain three monosaccharides, fucose, galactose, and mannose. In addition, it also contains a certain amount of uronic acid and sulfate groups, the sulfation mainly occurred at C2 or C4 of the fucose residue and C2, C4 or C6 of the galactose residue (14–16). Through methylation analysis and NMR analysis of purified components, it was shown that SFSP had the following repeating units of →2)-α-D-Man-(1 → 4)-β-D-GlcA-(1 → , → 3)-β-L-Fuc-(1 → 3,4)-β-L-Fuc-(1 → 3,4)-β-L-Fuc-(1 → and →3,4)-β-L-GlcA-(1→, →4)-β-L-Xyl-(1→, →4)-β-L-Gal-(1→, →3,6)–β-L-Manp-(1→ (14, 17, 18). Modern pharmacological research revealed that SFSPhas multiple bioactivities. SFSP could promote the immune responses in macrophages via inducing the CD14/IKK/NF-κB and P38/NF-κB signaling pathways (19), and it could have the ability to resist oxidative stress damage induced by lipopolysaccharides in cells and inhibit tumor angiogenesis (4). Moreover, Cheng reported that SFSP could alleviate HFD-induced early fasting hypoglycemia and regulate the structure of gut microbiota (20). However, so far, there has been relatively little research on the digestion and fermentation characteristics of sulfated polysaccharides from *Sargassum fusiforme*.

The present study aimed to reveal the digestion behavior and fermentation characteristics of SFSP in the digestion tract. It demonstrated the digestion behavior of SFSP by monitoring the change of molecular weight and the release of free monosaccharides in simulated digestion. Then the sugar content, monosaccharide composition and the production of short chain fatty acids were monitored in the fermentation of SFSP by human gut microbiota. Moreover, the regulation effect of SFSP microbiota composition was evaluated and the changes in metabolites of gut microbiota were also determined by ultra performance liquid chromatography-time of flight mass spectrometry (UPLC-TOF-MS). The findings of the present study could promote the exploration and utilization of SFSP and *S. fusiforme* in industries of foods and pharmaceuticals.

## 2 Materials and methods

### 2.1 Materials and chemicals

Brown seaweed *Sargassum fusiforme* was collected in July 2019 from Wenzhou, China. The dextrans (1, 5, 25, 50, 41 kDa) and monosaccharides (D-glucose, D-galactose, D-mannose, D-rhamnose, D-xylose, L-fucose, D-arabinose, D-glucuronic acid, D-galactose acid, D-Galactosamine, D-Glucosamine) used as standards for High performance liquid chromatography (HPLC) were purchased from sigma Chemical Co., Ltd. (St. Louis, MO, United States). The SCFA_S_ (Acetic acid, propionic acid, butyric acid), digestive enzymes (Gastric lipase, pepsin, pancreatin, and trypsin) and ammonium acetate were purchased from Aladdin Industrial Inc. (Shanghai, China). High performance liquid chromatography grade solvents including acetonitrile, chloroform, methyl alcohol and formic acid were purchased from Spectrum Chemical (New Brunswick, Canada). Other analytical grade chemicals were purchased from Sinopharm Chemical Reagent Co., Ltd. (Shanghai, China).

### 2.2 Preparation of SFSP

The preparation of SFSP was performed according to the previously reported method with some modifications (5). Briefly, the seaweed *Sargassum fusiforme* was washed, air-dried, smashed and mixed with deionized-water at 50°C for 30 min and the ratio of solid to liquid was 1: 20. The enzymatic hydrolysis process was implemented by using 1% cellulase, pectinase and papain (4: 1: 1, 620 U/g) at 50°C for 4 h. Then the solution was heated to 98°C to inactivate enzymes. After the centrifugation at 3000 rpm/min for 10 min, the supernatant was collected and concentrated to 1/10 of the original volume. Then a four-step ethanol precipitation was conducted to separate SFSP. Briefly, Firstly, ethanol was added to achieve a final proportion of 20%, and after standing for a night, the precipitate which mainly contained alginate was removed by configuration (10,000 rpm, 15 min, 4°C); Secondly, then the supernatant was collected and mixed with ethanol to achieve a final ethanol proportion of 60%, and the precipitate was collected by standing for a night and configuration (10,000 rpm, 15 min, 4°C); Thirdly, the precipitate was dissolved in hot water in a ratio of 1:10 (*m/v*), and then ethanol precipitation method was conducted again with an ethanol proportion of 30% to remove the residue alginate Fourthly, more ethanol was added to achieve a final proportion of 70%, and the precipitate was collected after configuration (10,000 rpm, 15 min, 4°C). The precipitate obtained by the four-step ethanol precipitation was dissolved in water (1.5%, *m/v*) and mixed with 2% CTAB solution (4:1, *v/v*). Then the resulting precipitate was dissolved in 3 M KCl, and SFSP was precipitated by adding ethanol with the final ethanol proportion of 70%. Finally, the resulting precipitate was dissolved in the deionized-water, dialyzed for 3 days to remove the salt, and lyophilized to obtain SFSP powder.

### 2.3 Characterization of SFSP

Total carbohydrate and uronic acid content in SFSP were measured using the phenol-sulphuric method and m-hydroxydiphenyl method (21, 22). Quantitative analysis of protein in SFSP was carried out according to the Bradford method (23). The sulfate group was quantified by the BaCl_2_-gelatin turbidimetric assay (24). The content of reducing sugar was measured by the method of DNS (25).

Fourier transform infrared spectroscopy (FTIR) assay of SFSP was determined via the Fourier transform infrared reflection (FTIR) Spectrometer (Perkin Elmer, Norwalk, United States) after pressed potassium bromide into tablet (2 mg of SFSP in 100 mg of KBr).

### 2.4 Simulated digestion

The fresh saliva was provided by four donors who had not been treated with antibiotic in the past 3 months. Then the saliva was mixed and centrifuged at 2500 g for 20 min and the supernatant was collected and used as oral digestion juices. 4 mL SFSP solution (2 mg/mL) was mixed with equal volume of oral juice for digestion in the water bath at 37°C for 2 h. The mixture of 4 mL deionized water and 4 mL oral digestive juice was used as blank control. Samples were collected at 0 h, 0.5 h, 1 h and 2 h and the enzyme was inactivated with a boiling water bath for 10 min for further analysis.

For simulated gastric and intestinal digestion, the simulated gastric juice and intestinal juice were prepared as previously described (26). The mixtures of 6 mL SFSP solution (4 mg/mL) and 6 mL gastric juice, and 6 mL deionized water and 6 mL gastric juice were subjected to simulated gastric digestion at 37°C and 150 rpm for 6 h in a constant temperature shaker. Samples were collected at 0 h, 1 h, 2 h, 4 h and 6 h and the enzyme was eliminated with a boiling water bath for 10 min. After gastric digestion, 4 mL solution was collected, neutralized with 1 M NaHCO_3_, and mixed with 4 mL intestinal digestive juice. Then the simulated intestinal digestion was performed with the constant temperature shaker for 6 h at 37°C and 150 rpm. Finally, samples of intestinal digestion were collected at 0 h, 1 h, 2 h, 4 h and 6 h and the enzyme was inactivated with boiling water for 10 min bath treatment. The molecular weight distribution and the released monosaccharides of samples at different times were measured according to the HPLC methods as we previously described (7).

### 2.5 *In vitro* fermentation of polysaccharides

Fecal samples were collected from 4 healthy volunteers (two males and two females, 18 ~ 24 years old) who had no history of intestinal diseases during last 3 months. Four equal fecal samples were mixed and dissolved in the saline solution containing 0.5 g/L cysteine-HCl (10%, *w*/*v*). 300 mL of the basal growth medium for *in vitro* fermentation was prepared by adding 0.6 g peptone, 0.6 g yeast extract, 0.006 g hemin, 0.15 g L-cysteine, 0.15 g bile salts, 0.03 g NaCl, 0.012 g K_2_HPO_4_, 0.012 g KH_2_PO_4_, 0.003 g MgSO_4_, 0.003 g CaCl_2_, 0.6 g NaHCO_3_, 0.3 mL resazurin solution (1%, *w*/*v*), 0.6 mL Tween-80, and 3 μL vitamin K. 500 mg SFSP was dissolved in 50 mL the basal growth medium and autoclaved for the next *in vitro* fermentation. 1.5 mL of fecal slurry was added to the culture medium containing polysaccharides and the basic culture medium as SFSP group and blank control group (CON), respectively. Each group had triple parallel experiment. Then, SFSP and CON group were transferred to an anaerobic incubator and fermented at 37°C for 48 h. The fermentation products of 0 h, 12 h, 24 h, and 48 h were collected and put into ice water for 5 min. Then these samples were centrifuged at 8000 g for 10 min and the supernatants were used for the further study.

### 2.6 Determination of pH and short chain fatty acids (SCFAs)

The pH values of supernatant samples were measured by a pH meter (S21 Seven compact, Mettler-Toledo instrument Co., Ltd., Shanghai, China). The contents of SCFAs were determined by a reported HPLC method with some modifications. In brief, 30 μL 10% sulfuric acid was added into 0.5 mL of the filtrate samples, and 2 mL ethyl ether was then added into the mixture to extract SCFA_S_ for 15 min. After centrifugation (3,500 rpm, 10 min), the supernatant was collected and alkalized by adding 500 μL 1 M NaOH. Then the resulting aqueous phase was acidified with 10% sulfuric acid again. The aqueous samples were analyzed on HPLC system (e2695, Wasters, Milford, United States) equipped with a Silgreen ODS C-18 column (250 × 4.6 mm, 0.5 μm) and a photodiode array detector (PDA). The operating parameters of HPLC were as follows: column oven temperature, 30°C; mobile phase A, phoric acid solution (0.025%, *v*/*v*); mobile phase B, acetonitrile; the mobile phase ratio, 95: 5; flow rate, 1.0 mL/min; detector wavelength, 205 nm; and injection volume, 10 μL.

### 2.7 Analysis of gut microbiota

After *in vitro* fermentation of 48 h, bacterium cells of SFSP and CON groups were separated by centrifugation at 8000 g for 10 min from fermentation broth. The total bacterial DNA in each group was extracted by the Power Fecal DNA Isolation Kit (MO BIO, Carlsbad, USA). Agarose gel electrophoresis method was used to evaluate the DNA quality. The V3 regions of 16S rRNA was amplified with universal primers 515F and 806R by PCR assay. The QIAquick Gel Extraction Kit was used for purification of PCR products and then sequenced by the Illumina HiSeq 2,500 platform. Bioinformatics classification of species in the different levels were based on the operational taxonomic units (OTUS) and the sequences with similarity of ≥97% were classified into an OTU unit individually performed by RDP classifier Bayesian algorithm at Usearch (version 7.0 http://drive5.com/uparse/). The biological classification information of each operational taxonomic unit (OUT) unit was obtained by matching the Silva database (http://www.arb-silva.de).

### 2.8 Analysis of microbial metabolites

Micromolecules produced by microbial fermentation were extracted by adding acetonitrile to 70%. The precipitate was removed by centrifugation (10,000 g, 10 min) and the supernatant cultivated with 0.22 μm filter was analyzed for metabolites. Microbial metabolites detection was performed by using UPLC- MS. Separation was carried out on an UPLC system (Nexera LC-30A, Kyoto, Japan) equipped with an Xselect HSS T3 column (100 × 2.1 mm, 2.5 μm, Waters). Mobile phases A and B were water/acetonitrile (95:5, *v*/*v*) with 0.1% formic acid and acetonitrile, respectively. The gradient elution was as follows: 0 min 15% B; 0 ~ 5 min 20% B; 5 ~ 7 min 20% B; 7 ~ 14 min 100% B; 14 ~ 16 min 100% B; 16 ~ 16.1 min 20% B; 16.1 ~ 21 min 20% B. Untargeted metabolites mass detection was performed on a Triple TOF 5600 (AB SCIEX, Milwaukee, USA) in the electrospray ionization (ESI) (+) mode. The dry gas was 10 L/min at 350°C. The declustering potential, the collision energy, and the capillary voltage were 40 V, 10 V, and 5,500 V. Information dependent acquisition (IDA) Experiment mode was selected and the cycle time was 1.0011 s for 1,259 cycles during 21.007 min. Tuning Mix solution (AB SCIEX, Milwaukee, United States) was applied to the instrument calibration and formate solution of 10 mM sodium was used for auto internal calibration.

### 2.9 Statistical analysis

The data were reported as mean ± standard error of mean (SEM) using Student’s t-test for comparison or one-way ANOVA tests for multiple comparisons by SPSS version 9.0 software. The principal component analysis (PCA) was performed by MetaboAnalyst 4.0.

## 3 Results and discussion

### 3.1 Characterization of SFSP

**Figure 1:**
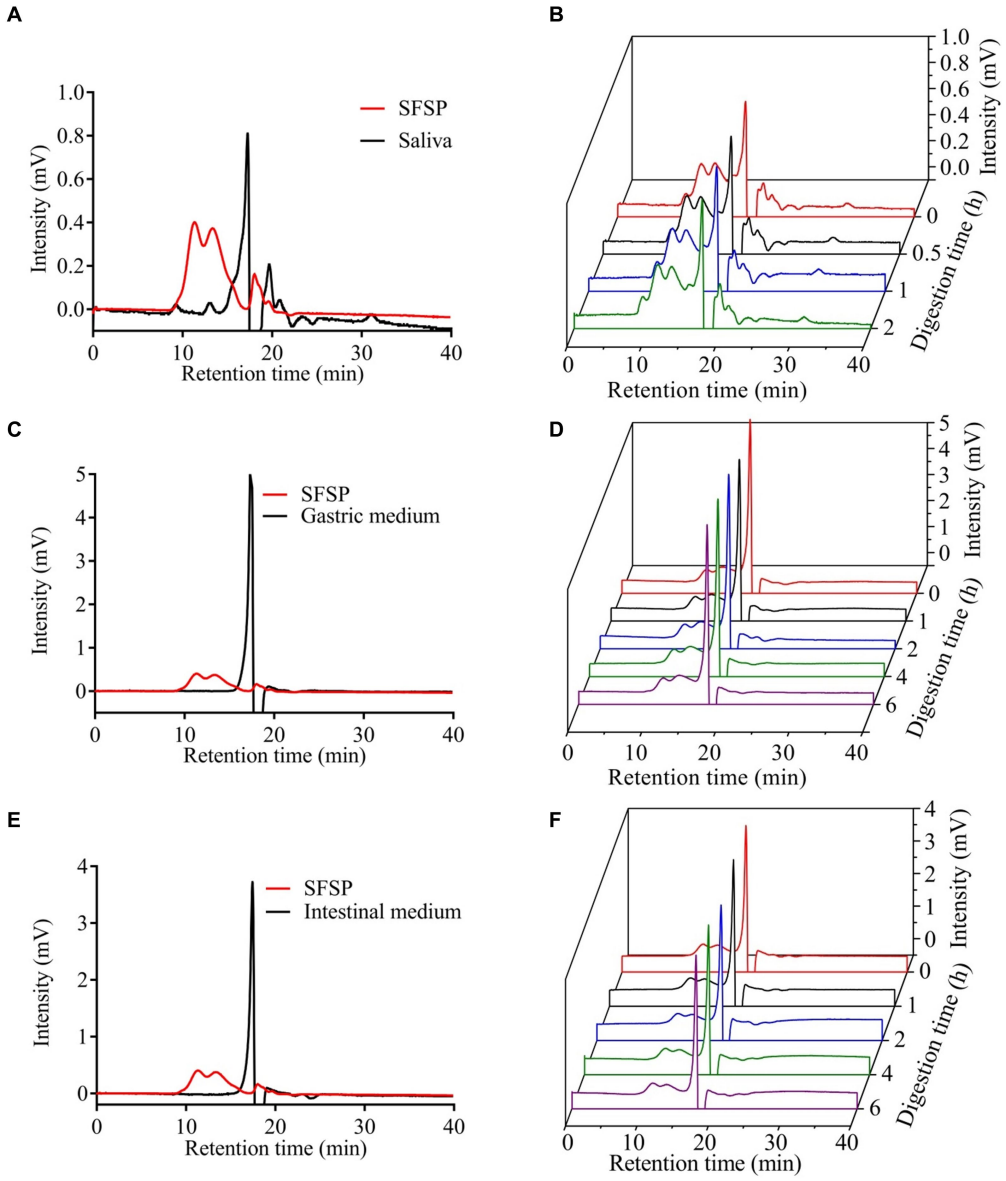
The retention times of saliva **(A)**, gastric medium **(C)**, intestinal medium **(E)** and the change of the Mw of SFSP at different times during simulated mouth **(B)**, stomach **(D)** and small intestine **(F)** digestion.

SFSP was obtained from *Sargassum fusiforme* by enzymatic hydrolysis and stepwise ethanol precipitation followed by the quaternary ammonium salt precipitation. The total carbohydrate and uronic acid contents of SFSP were 39.25 ± 1.01% and 13.72 ± 0.30%, respectively. SFSP had a significantly higher sulfate content (34.39 ± 2.01%) than previously reported (14, 17) which could be attributed to the application of CTAB which could selectively adsorb acidic polysaccharides. The monosaccharide composition analysis showed that SFSP mainly contained fucose, galactose, mannose, rhamnose, glucuronic acid, glucose and xylose in a ratio of 55.39: 24.01: 8.09: 1.23: 9.07: 0.7: 1.51. The proportions of fucose and galactose in SFSP were similar to those of the polysaccharide extracted from *Sargassum fusiforme* previously by Cheng et al. (20), but the contents of glucuronic acid and the other neutral monosaccharides were different from Cheng’s report. Moreover, a small amount of protein (0.88 ± 0.05%) was detected in SFSP. From the high-performance gel permeation chromatography (HPGPC) chromatogram of SFSP in Figure 1A, two peaks were observed except for the salt peak. Based on the dextran standards, the molecular weights (Mw) for fractions I and II were calculated to be 526.9 kDa and 106 kDa. In addition, The FTIR spectrum (Figure 1B) showed the typical absorption peaks of polysaccharides. The signals at 1654 cm^−1^ were assigned to symmetric stretching vibration of C=O due to the uronic acid in SFSP. The strong signals around 1,250 cm^−1^ were attributed to the O=S=O asymmetric stretching vibration from sulfate indicated SFSP was a highly sulfated polysaccharide (27).

### 3.2 Possible change of SFSP during *in vitro* digestion

**Figure 2:**
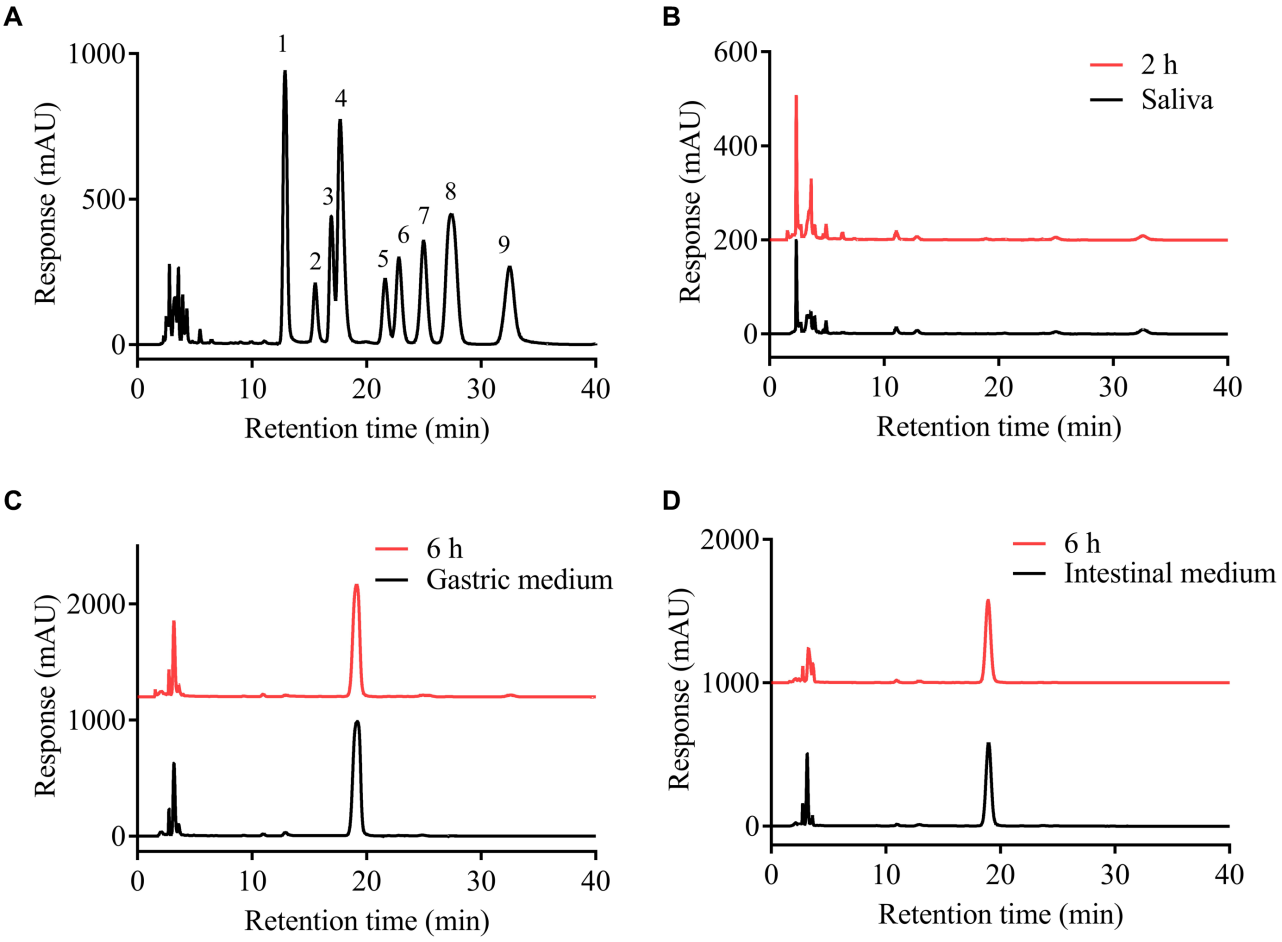
High performance liquid chromatograms of PMP derivatives of the mixed standard monosaccharides **(A)** and the analysis of the free monosaccharides released from SFSP at different times during simulated mouth **(B)**, stomach **(C)** and small intestine **(D)** digestion. 1, mannose; 2, rhamnose; 3, glucuronic acid; 4, galacturonic acid; 5, glucose; 6, galactose; 7, xylose; 8, arabinose; 9, fucose.

The upper digestive system including saliva, gastric and small intestinal digestion with enzymes and acidic environment has the hydrolysis ability for some carbohydrates (28). The simulated digestion process of SFSP was monitored by detecting the changes of Mw, reducing sugars and free monosaccharides of digested samples. As shown in Figures 1A,B, the retention times of fractions I and II in HPGPC were not changed during digestion. This indicated that the treatment of saliva had no effect on SFSP. Furthermore, the gastrointestinal digestion was performed subsequently by the simulated physiological environment and a mixed enzyme system in our study. As shown by the chromatogram peaks in Figures 1C–F, the two major fractions of SFSP remain unchanged under both gastric and small intestinal digestion conditions. Moreover, no free monosaccharide was detected after saliva, gastric and small intestinal digestion (Figure 2). These results all indicated that SFSP could not be degraded in the upper digestive system. Sulfated polysaccharides from *Ascophyllum nodosum*, another kind of brown seaweed, also exhibit resistance to digestion as reported in previous study (29). But a report on research from *Laminaria japonica* sulfated polysaccharides showed that, it could resist the digestion of saliva and simulated gastric juice, but it was degraded in simulated small intestine juice (30).

### 3.3 Utilization of SFSP by human gut microbiota during *in vitro* fermentation

**Figure 3:**
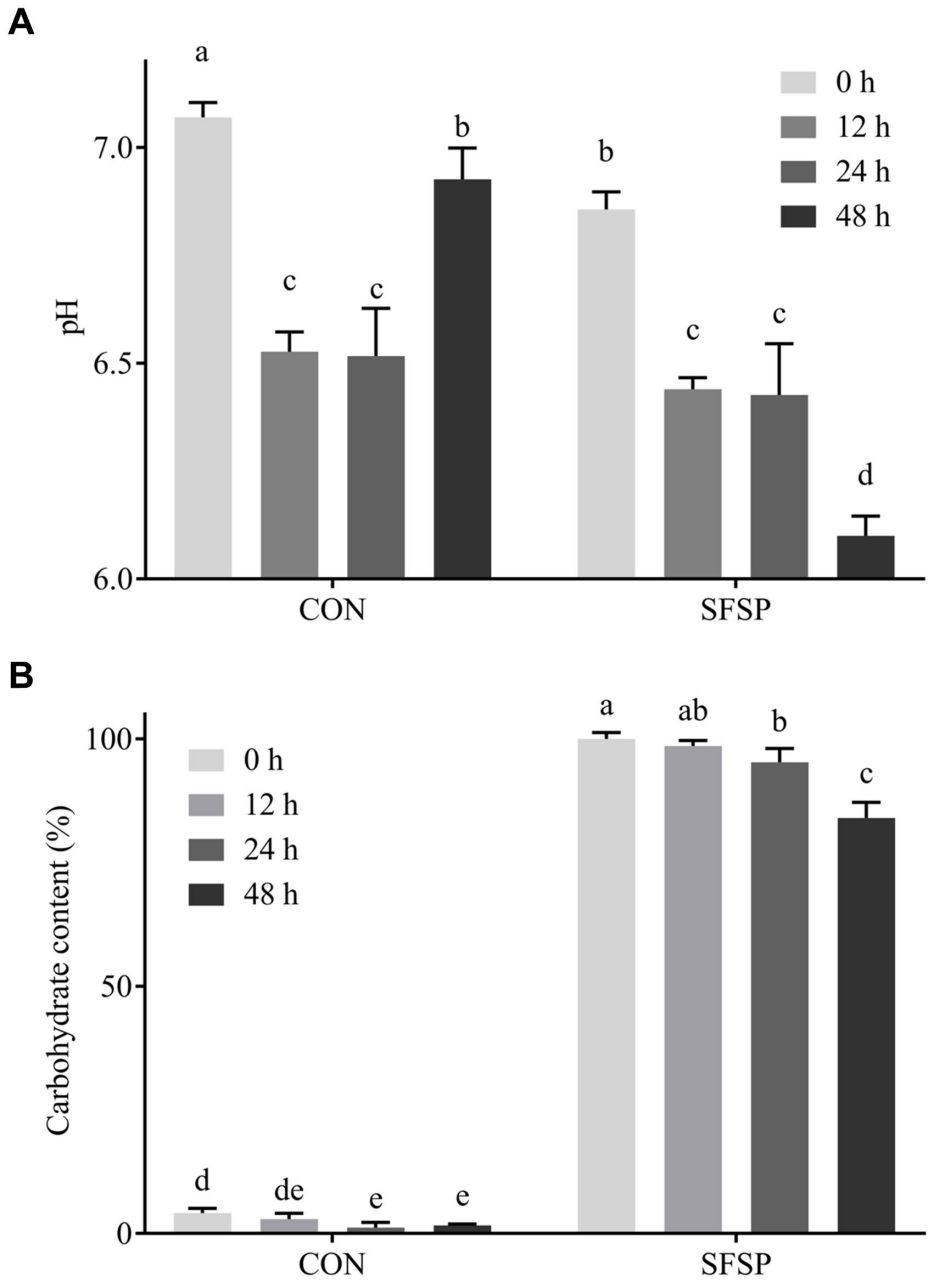
Changes of pH value **(A)** and total carbohydrate **(B)** during 48 h *in vitro* fermentation.

Undigested polysaccharides are possibly utilized by gut microbiota, and the SCFAs were considered as their major metabolites (8). So carbohydrate content, pH value, and SCFA contents of SFSP fermentation broth were monitored. As shown in Figure 3B, the total carbohydrate content of SFSP group decreased gradually, and after 48 h of *in vitro* fermentation, 16% of total carbohydrate was consumed in SFSP group. The pH value changes as a fundamental feature of fermentation processes were monitored at different times (Figure 3A). The initial pH values of SFSP and CON were 7.07 ± 0.03 and 6.86 ± 0.03, respectively, and the difference may be attributed to the uronic acid in SFSP structure (28). After fermentation for 12 h, the pH values of SFSP and CON evidently decreased to 6.53 ± 0.03 and 6.44 ± 0.02, respectively. Thereafter, the pH of SFSP and CON groups had tended to be stable during 12–24 h fermentation, but then the pH value of SFSP group began to decrease and reached to 6.09 ± 0.04 at 48 h. As shown in Table 1, SCFA contents were consistent with pH values observed at these time points, which confirmed that SFSP were utilized by gut microbiota to produce SCFAs. The decrease in pH value and the increase in short chain fatty acid content may be a common characteristic of the fermentation of sulfated polysaccharides by gut microbiota, and these results were also observed in the fermentation of sulfated polysaccharides *Sargassum thunbergii* and *Laminaria japonica* (31, 32).

**Table 1:** Concentrations of SCFAs in fermentation solutions at different time points of fermentation *in vitro.*

Short chain fatty acids	Samples	Fermentation time (h)			
		0	12	24	48
Acetic acid	CON	2.72 ± 0.26^A^	5.66 ± 0.50^aB^	8.70 ± 0.78^aC^	10.20 ± 0.36^aD^
	SFSP		25.04 ± 0.94^bB^	28.37 ± 1.23^bC^	29.53 ± 0.40^bC^
Propionic acid	CON	1.36 ± 0.09^A^	4.33 ± 0.38^aB^	6.45 ± 0.05^bC^	7.00 ± 0.49^aC^
	SFSP		4.61 ± 0.36^aB^	5.86 ± 0.26^aC^	9.77 ± 0.52^bD^
Butyric acid	CON	ND	4.31 ± 0.19^A^	7.43 ± 0.25^bB^	4.47 ± 0.13^aA^
	SFSP		ND	3.51 ± 0.16^aA^	4.32 ± 0.47^aB^

The different lowercase letters mean significant difference (*P* < 0.05) for each SCFA existed for different treatment groups. Different capital letters indicate significant differences (*p* < 0.05) for each SCFA among different time points. ND is not detected.

**Figure 4:**
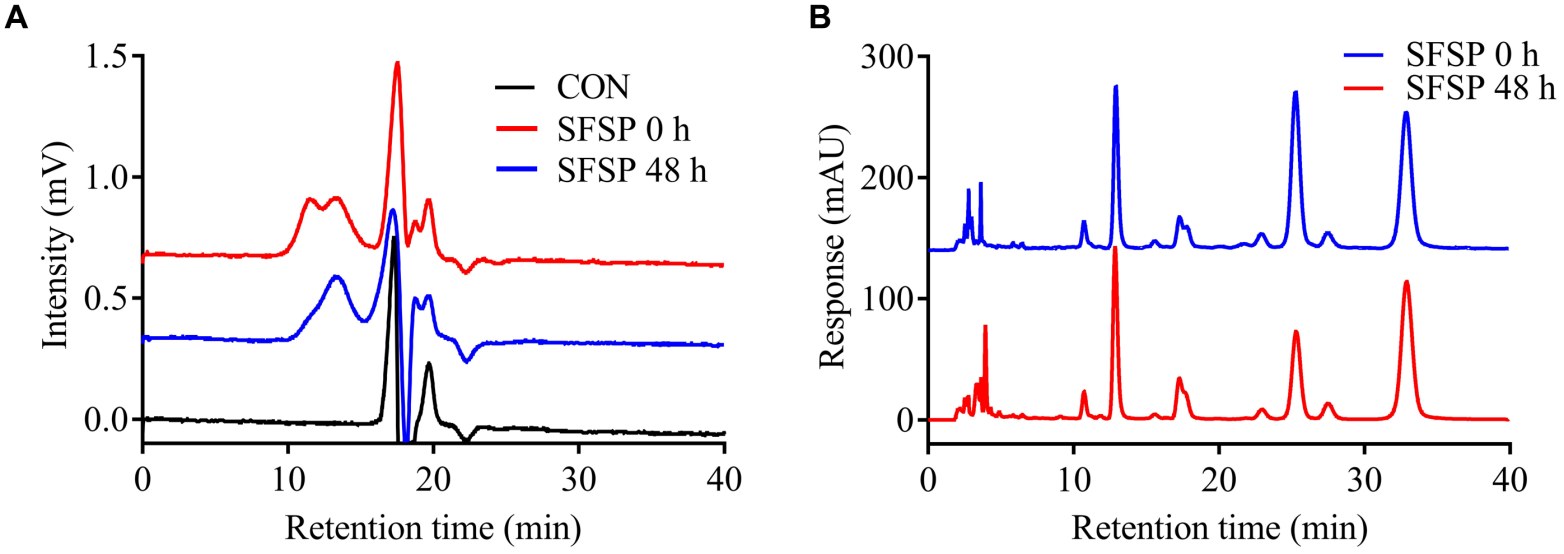
Analysis of the Mw of SFSP **(A)** and monosaccharides released from SFSP **(B)** after 48 h *in vitro* fermentation.

It has been reported that different fractions of dietary polysaccharides might be selectively utilized by gut microbiota in fermentation (28). In addition, the fermentation characteristics of polysaccharides were greatly related to their component monosaccharides (33). Therefore, in order to reveal the utilization preferences of the gut microbiota for SFSP fractions, HPGPC profiles and monosaccharide compositions of the polysaccharides in the fermentation solutions were measured in the present study. The molecular weight distribution of SFSP at 0 and 48 h *in vitro* fermentation were shown by HPGPC in Figure 4A. Interestingly, the peak of fraction I disappeared after 48 h fermentation while peak of fraction II did not change obviously. Furthermore, the monosaccharide compositions of the polysaccharides in the fermentation solutions at 0 h and 48 h were measured and compared (Figure 4B). Compared to that of original SFSP (0 h), after 48 h fermentation, the molar ratio of galactose to fucose of residual polysaccharides decreased from 1: 2.31 to 1: 5.27 while the ratios of other monosaccharides (manmose and glucuronic acid) to fucose almost remained unchanged. These results indicated that galactose in SFSP was prone to be utilized or degraded by human gut bacteria. It could be inferred that Fraction I, which was utilized in fermentation, was mainly composed of galactose, and it was the effective prebiotics in SFSP for gut microbiota. Fraction II containing Fuc as the major monosaccharide component was resistant to the fermentation, and this phenomenon has also been found for some fucoidan and galactofucan from brown algae (34).

### 3.4 Effects of SFSP fermentation *in vitro* on gut microbiota

**Figure 5:**
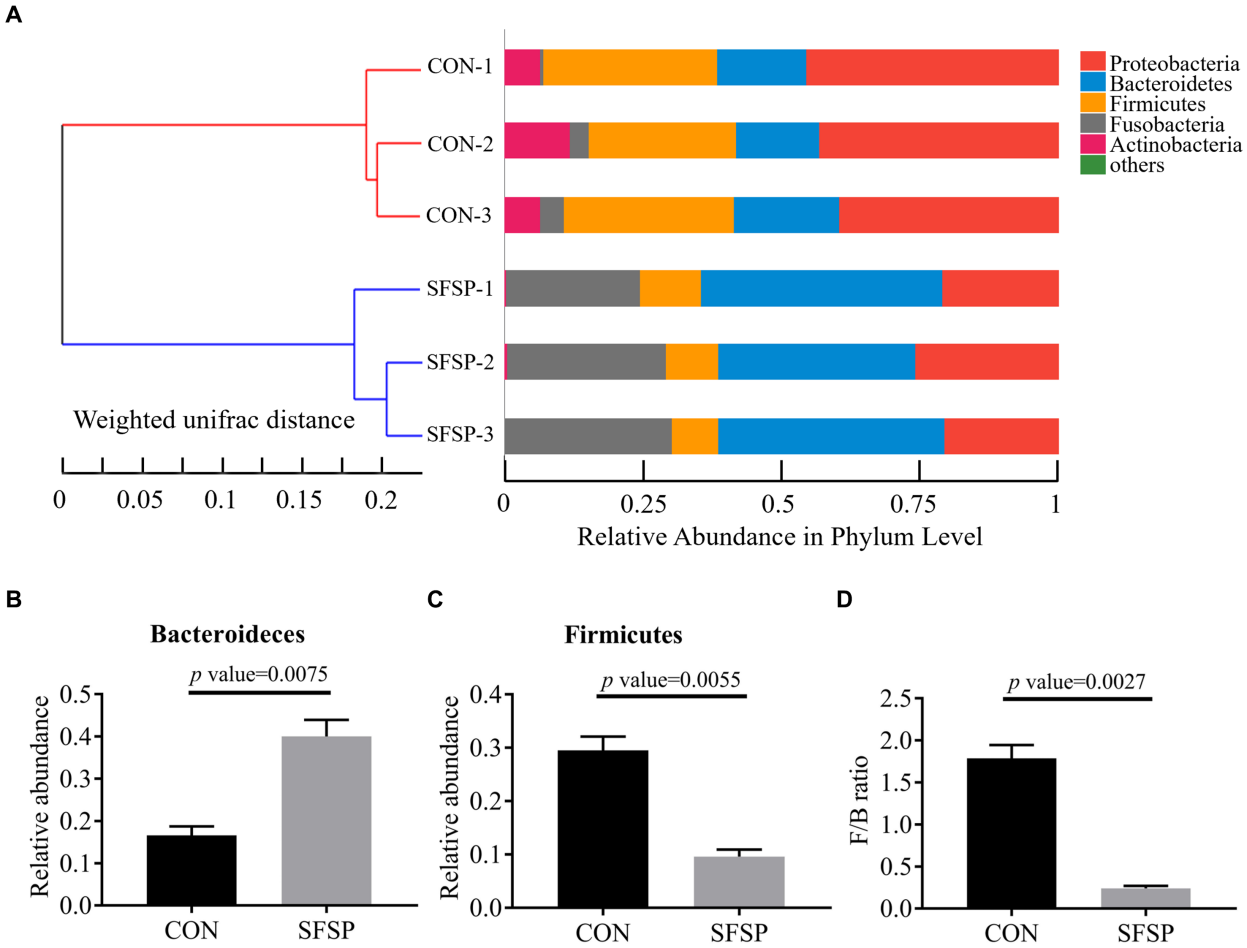
UPGMA analysis **(A)**, relative abundance of Bacteroidetes **(B)**, relative abundance of firmicutes **(C)**, F/B ratio **(D)**. Data were presented as mean ± SEM (*n* = 3).

To reveal the regulation effect of SFSP on the gut microbial community, the bacterial 16S rRNA V3 regions amplicon sequencing was performed to compare the CON and SFSP groups after 48 h *in vitro* fermentation (5). As shown in Figure 5A, at the phylum level, all the samples mainly consisted of Firmicutes, Bacteroidetes, Proteobacteria, Fusobacteria, and Actinobacteria, but the hierarchical cluster analysis using unweighted pair-group method with arithmetic means (UPGMA) showed the distinction of microbial community composition between the two groups. Furthermore, as shown in Figures 5B,C, compared with the CON group, SFSP significantly reduced the abundance of Firmicutes and promoted the growth of Bacteroidetes (*p* < 0.05), resulting in the significantly heighted ratio of Firmicutes phylum to Bacteroidetes phylum (F/B) in SFSP group (*p* < 0.05) in Figure 5D. Our results are similar to those of a recent study on the fermentation of sulfated polysaccharides from *Laminaria japonica*, which the high molecular weight component of sulfated polysaccharides from *Laminaria japonica* significantly down regulated the value of F/B (35). This suggests that molecular weight may be an important factor in regulating gut microbiota structure. It has been well documented that obese individuals generally have higher F/B ratios (36), while the restoration of the F/B ratio by total fecal microbiota transplantation or dietary fiber intervention could prevent weight gain (37, 38). Our findings also indicate that SFSP could also prevent obesity by decreasing the F/B ratio.

**Figure 6:**
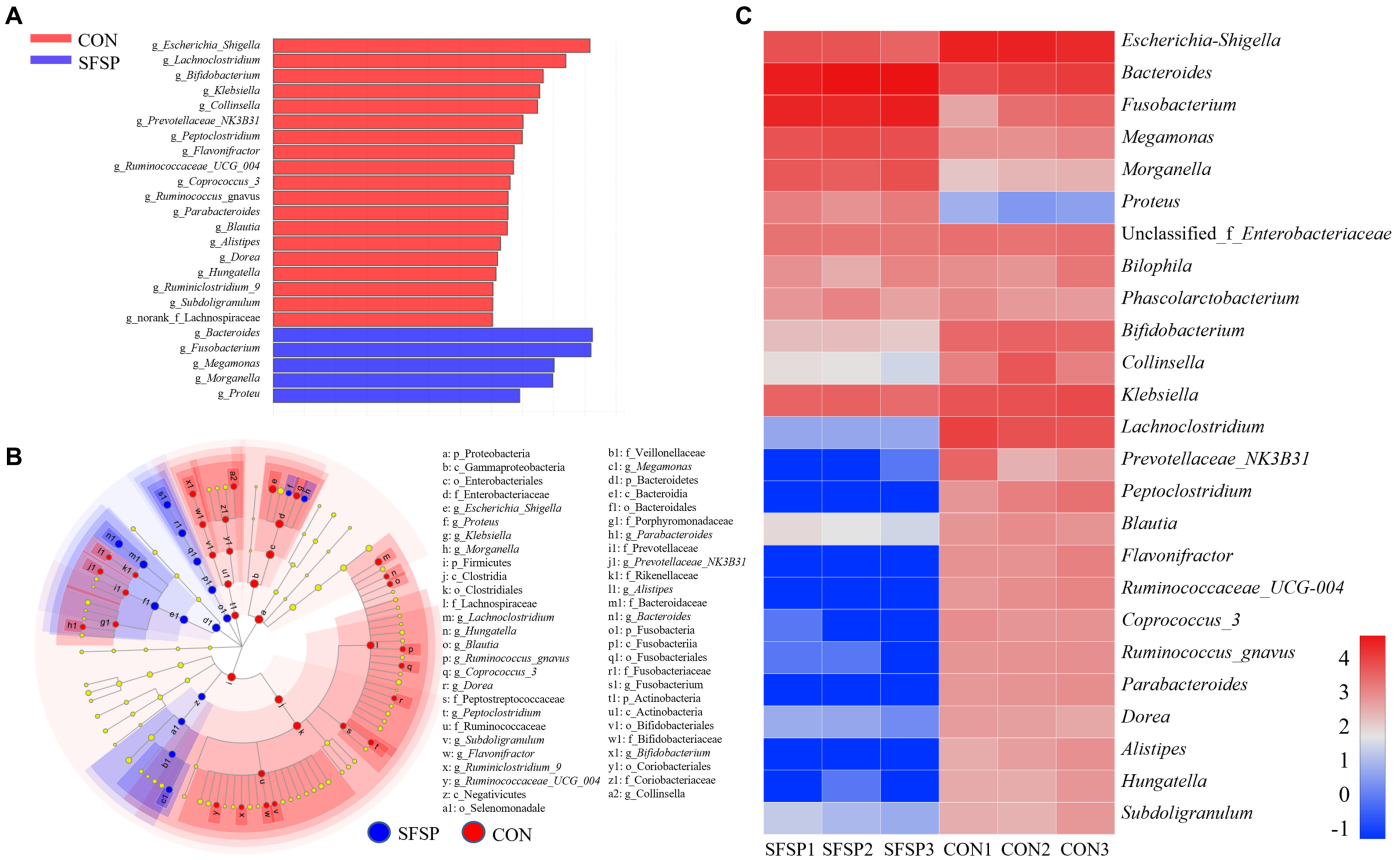
LDA score **(A)**, heatmap of top 25 dominant genera **(B)** and LEfSe analyses **(C)** between SFSP group and CON group (*n* = 3, LDA score > 3).

Top 25 genera of bacteria found in the samples were shown in Figure 6B, and *Escherichia_Shigella*, *Bacteroides*, *Fusobacterium*, *Megamonas*, *Morganella*, *Proteus*, *Enterobacteriaceae*, *Bilophila*, *Phasolarctobacterium*, *Bifidobacterium*, *Collinsella*, *Klebsiella*, *Lachnoclostridium* (%) were the main genera after *in vitro* fermentation. In order to identify the altered genus attributed to the microbiota composition, linear discriminant analysis effect size (LEfSe) was carried out between CON and SFSP groups from the Phylum level to genus level as shown in Figure 6C. It was obvious that SFSP showed inhibitory effect on many genera from Clostridia, and greatly promote the growth of *Bacteroides* resulting an obviously increase of the Bacteroidetes phylum. On the basis of the Linear Discriminant Analysis (LDA) score (higher than 3) in Figure 6A, the addition of SFSP resulted in an increase in a total of 5 genera (*Bacteroides, Fusobacterium*, *Megamonas, Morganella*, *Proteu*) and lowered the abundances of 19 genera mainly including *Escherichia_Shigella, Lachnoclostridium, Bifidobacterium, Klebsiella,* and *Collinsella*.

It was obvious that SFSP feeding could significantly promote the proliferation of *Bacteroides*. Previous studies have shown that many species of *Bacteroides* contain a variety of hydrolases to degrade polysaccharides, such as *B. thetaiotaomicron* and *B. uniformis*, and they could synthesize glucosidases for fructan and agarose fermentation (39, 40). In addition, recent studies have shown that the increase of some *Bacteroides*, such as *B. fragilis*, could prevent the chronic colitis and colon tumors (41). *Fusobacterium*, eg. *F. gonidiaformans* and *F.* var*ium*, could produce butyric acid by the utilization of protein in gut on the basis of the lysine pathway (42). *Megamonas*, eg. *M. rupellensis*, was the acetic acid and propionic acid producer (43). These two kinds of genera might regulate host health as the SCFAs producers. *Escherichia_Shigella*, eg. *E. coli*, was considered as opportunistic pathogen might cause an intestinal infection (44). *Lachnoclostridium* spp. had been reported to be significantly enriched in adenoma and DSS-induced colitis model (45). *Collinsella* had been described as a beneficial genus in some studies (46). But more reports showed that *Collinsella* had a strong correlation with a variety of diseases, such as nonalcoholic steatohepatitis, type 2 diabetics and atherosclerosis (47, 48). *Klebsiella* was regarded as typical opportunistic pathogenic bacteria, such as *Klebsiella pneumonia*, and it was the major pathogen in human pneumonia. It had been reported that *Klebsiella pneumonia* could induce fatty liver in mice by producing excessive alcohol (49). These results all illustrated that SFSP could be utilize by the *Bacteroides* and inhibited the growth of intestinal pathogens.

### 3.5 UPLC-MS/MS reveals the effect of SFSP on the gut microbiota metabolite signatures

**Figure 7:**
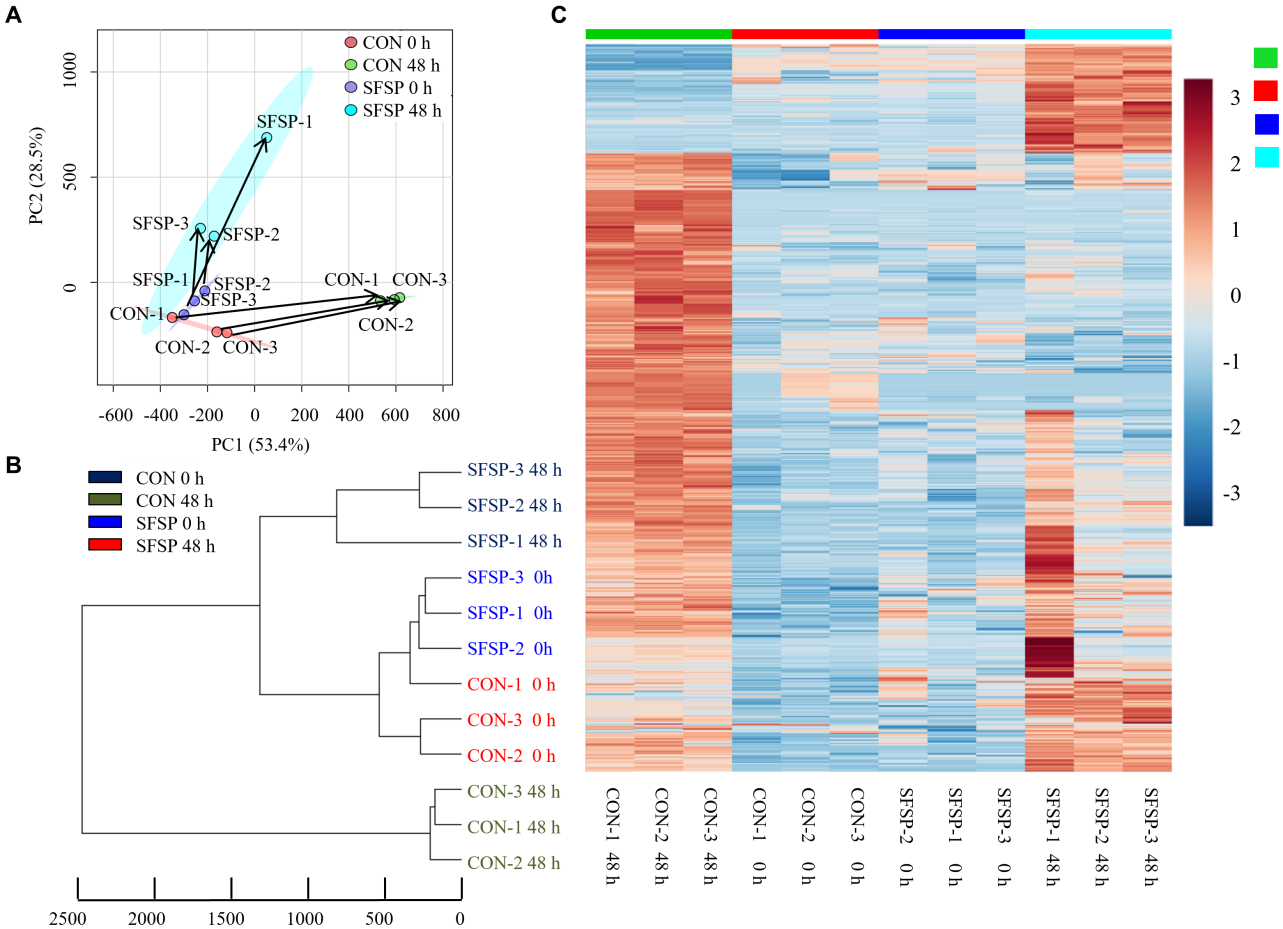
PCA **(A)**, UPGMA **(B)** analysis and heatmaps **(C)** of the differential metabolite profiles between SFSP group and CON group\with fold change >2 or < 0.5. Data are expressed as mean ± SEM (*n* = 3) and *p* < 0.05 by the Student’s *t*-test.

A UPLC-MS/MS-based method was performed to investigate the small molecules produced by the gut microbiota using an *in vitro* fermentation modal. Principal component analysis (PCA) of metabolites revealed the substrate-specific clustering for both CON and SFSP samples (Figure 7A). At the starting point of fermentation, all these samples were close to each other in the direction of PC1 or PC2, indicating that the addition of SFSP had no effect on the small molecular components of the medium. After 48 h of fermentation, the gut microbiota metabolites of CON group changed significantly in PC1 while that of the SFSP changed mainly in PC2. Based on the intensities of the detected features in samples, the differentiation between gut microbiota metabolic capacity was also analyzed by hierarchical clustering (Figure 7B). A total of 945 differentiated metabolites were detected in the two groups at 0 h and 48 h. (Figure 7C) These results indicated that small molecular metabolites produced by microbiota were significantly changed with the utilization of SFSP.

Confirming the chemical identity of the detected m/z values is the major obstacle in metabolomic studies because there are few databases and kinds of metabolites that can be used to characterize metabolites. Therefore, we selected a metabolite database which contains MS, MS/MS and isotopic molecular information of more than 500 metabolites. We matched the information of 945 small molecule compounds produced by microorganisms with the compounds in the database, and 11 marker metabolites were identified as shown in Table 2 and chemical structure were drawn in Figure 2A. Among them, 7 compounds increased significantly and 4 compounds including trimethylamine and 7α-hydroxy-3-oxo-5β-cholanoic acid decreased significantly. These compounds are involved in the pathways of amino acid, lipid and secondary bile acid metabolism.

**Table 2:** Identification results of differential metabolites between the CON group and the SFSP group.

	Adduct	Metabolite	Elemental composition	Fold change	*m/z*	Retention time (min)	Changing trends
1	(M + H) ^+^	Trimethylamine	C_3_H_9_N	4.6544	60.0807	0.63	**↓**
2	(M + H) ^+^	1,3-Dimethyluracil	C_6_H_8_N_2_O_2_	84.572	141.0659	0.7	**↓**
3	(M + H) ^+^	Pantothenic acid	C_9_H_17_NO_5_	0.0251	220.1176	2.82	**↑**
4	(M + H) ^+^	L-Valine	C_5_H_11_NO_2_	0.0146	118.0862	0.75	**↑**
5	(M + H) ^+^	Uracil	C_4_H_4_N_2_O_2_	0.4323	113.0346	0.81	**↑**
6	(M + H) ^+^	Nicotinic acid	C_6_H_5_NO_2_	0.0052	124.0393	0.82	**↑**
7	(M + H) ^+^	2-Piperidinone	C_5_H_9_NO	10.989	100.0757	1.95	**↓**
8	(M + H) ^+^	Citrulline	C_6_H_13_N_3_O_3_	0.0003	176.103	0.64	**↑**
9	(M + H) ^+^	Phenylacetaldehyde	C_8_H_8_O	0.0024	121.0648	0.85	**↑**
10	(M + H) ^+^	Aminocaproic acid	C_6_H_13_NO_2_	0.0116	132.1019	0.85	**↑**
11	(M + H) ^+^	7α-Hydroxy-3-oxo-5β-cholan	C_25_H_38_O_4_	3.9796	391.2843	11.57	**↓**

Trimethylamine is produced by the choline utilization cluster (cutC) (50) of gut bacteria from choline, and it could be absorbed into the enterohepatic circulation where it could be converted to trimethylamine oxide by oxidases in the liver. Trimethylamine oxide has been proved to be related to arteriosclerosis (48). It has been reported that trimethylamine oxidation is related to the ratio of Bacteroidetes and Firmicutes which is consistent with the results of the present study (51). *Klebsiella* has been proven to produce choline lyase cutC (52) and in Section 3.4, we have stated that SFSP can reduce the abundance of *Klebsiella*. It can be speculated that dietary SFSP may regulate trimethylamine by changing the ratio of Bacteroides and Firmicutes as well as reducing the abundance of the cutC enzyme producing bacterium *Klebsiella*. 7α-hydroxy-3-oxo-5β- cholanoic acid is a secondary bile acid whose biological function is constantly being discovered, such as regulating serum glucose and triglyceride (53). Nicotinic acid and pantothenic acid belong to the vitamin B family and can regulate a variety of physiological metabolism. The gut microbiota has been proven to be a widespread provider of vitamins (54) Studies have shown that supplementing with vitamin B can reshape the gut microbiota structure of obese individuals and have anti-obesity effects (55). It can be inferred that SFSP induces the production of more vitamin B in the gut microbiota, which may help alter the microbiota structure and prevent obesity. Phenylacetaldehyde is an aromatic compound that is often detected in human urine. Prebiotics can up-regulate the content of phenylacetaldehyde *in vitro* (56). Uracil and its precursor 1.3-dimethyluracil belong to base compounds. Previous studies have shown that uracil could stimulate the differentiation of intestinal epithelial cells, but may also lead to excessive immune production of reactive oxygen species (57). Citrulline, a precursor of arginine, is considered to have the same activity as arginine. It has been reported that citrulline supplementation can maintain the integrity of intestinal epithelial cells, and it can also alleviate the intestinal disorders and insulin resistance (58, 59). In addition, we conducted a correlation analysis between differential microbial communities and differential metabolites (Figure 2B). The content of pantothenic acid in fermentation broth is positively correlated with *Proteus*, *Bacteroides*, *Megamonas*, *Fusobacterium*, and *Morganella*. There is a positive correlation between the content of 7α-Hydroxy-3-oxo-5β-cholan and phenylacetaldehyde. The content of aminocaproic acid and nicotinic acid is positively correlated with the abundance of *Flavonifractor*, *Prevotellaceae-NK3B31u*, *Coprococcus-3*, and *Lachnochlostrium*. Trimethylamine, the pathogenic factor of atherosclerosis, is also positively correlated with the abundance of *Blautia*, *Klebsiella*, *Ruminococcus_gnavus*, *Flavonifractor*, *Peptoclostridium* and *Ruminococcae_UCG-004*. *Klebsiella* and trimethylamine showed a strong correlation, which confirms the previous speculation that SFSP may improve human health by inhibiting pathogenic bacteria.

## 4 Conclusion

The present study demonstrated the digestion behavior and fermentation characteristics of SFSP in the simulated digestion tract environment. The results suggested that SFSP could not be digested and could reach the colon as prototypes. During the fermentation with human intestinal flora, the higher-molecular-weight fraction of SFSP was utilized, and the galactose content in monosaccharide composition decreased relatively, which indicates that SFSP fractions can be selectively utilized by human gut microbiota. At the same time, SFSP also changed the composition of intestinal microbiota such as increasing the abundance of Bacteroides and decreasing the abundance of Firmicutes. SFSP promoted the production of acetic, propionic and n-butyric acids significantly. Moreover, SFSP could down-regulate the contents of trimethylamine, piperidone and secondary bile acid and increase nicotinic acid, pantothenic acid and other organic acids. The characteristics of SFSP in the fermentation with human intestinal flora suggests its potential application as nutritional supplement to benefit human health.

## Data availability statement

The original contributions presented in the study are included in the article/Supplementary material, further inquiries can be directed to the corresponding author.

## Author contributions

LJ: Conceptualization, Data curation, Formal analysis, Investigation, Methodology, Project administration, Software, Supervision, Validation, Writing – original draft, Writing – review & editing. CS: Validation, Writing – review & editing. CA: Methodology, Writing – review & editing. CW: Methodology, Writing – review & editing. SS: Conceptualization, Formal analysis, Funding acquisition, Methodology, Project administration, Resources, Supervision, Visualization, Writing – review & editing.
