# Supplementary material for: Modulation effect of sulfated polysaccharide from Sargassum fusiforme on gut microbiota and their metabolites in vitro fermentation
Source: Front Nutr. 2024 May 1;11:1400063. doi: 10.3389/fnut.2024.1400063 (PMC11094809; doi:10.3389/fnut.2024.1400063)
Supplement: Supplementary file 1 [file Image_1.pdf]

Supplementary Material

1 Supplementary Figures

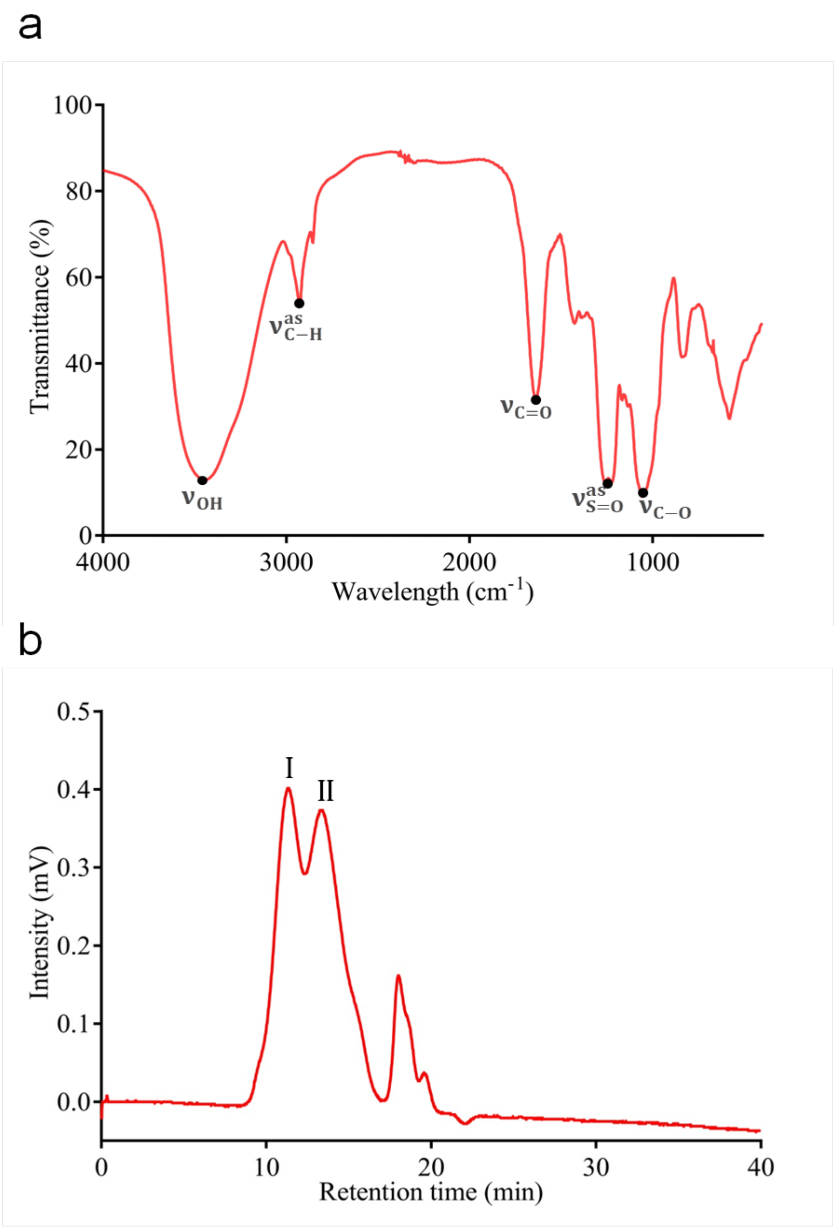

Supplementary Figure 1. FT-IR spectrum (**a**) and gel permeation chromatography (**b**) of SFSP.

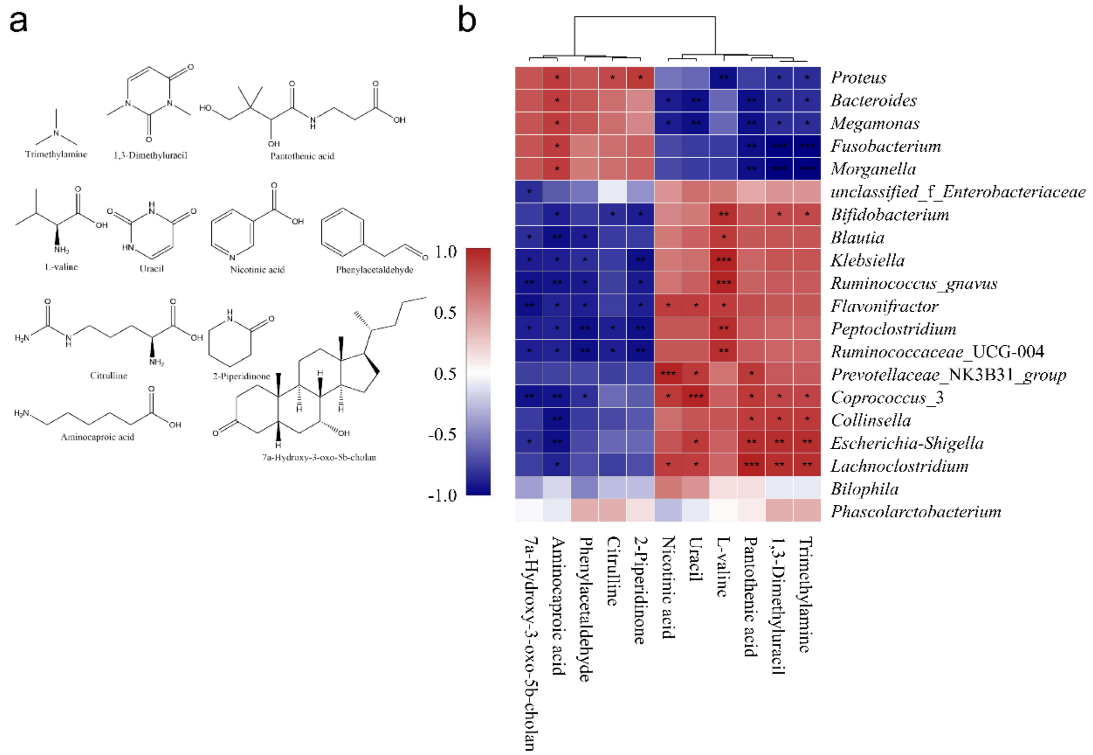

Supplementary Figure 2. Identified metabolites (**a**) and heatmap (**b**) of Spearman's correlation between the bacteria significantly differentially abundant with gut microbiota and differential metabolites. Significant correlations are marked by *p < 0.05, **p < 0.01 and ***p < 0.001..
